# TRENDS IN THE CLINICALPRESENTATION OF CELIAC DISEASE IN BRAZILIAN ADULTS SEEN IN THE LAST 50 YEARS IN A PRIVATE OFFICE

**DOI:** 10.1590/S0004-2803.24612025-118

**Published:** 2026-03-23

**Authors:** Lorete Maria da Silva KOTZE, Shirley Ramos da Rosa UTIYAMA, Luiz Roberto KOTZE, Fabiana Antunes ANDRADE, Renato NISIHARA

**Affiliations:** 1 Universidade Federal do Paraná, Curitiba, Paraná, Brasil.; 2 Universidade Positivo, Departamento de Medicina, Curitiba, Paraná, Brasil.

**Keywords:** Celiac disease, clinical presentation, diagnosis, doença celíaca, apresentação clínica, diagnóstico

## Abstract

**Background:**

Since the mid-20th century, celiac disease (CD) has evolved from being regarded as a predominantly pediatric disorder to one increasingly recognized across all ages. In Brazil, CD was initially underdiagnosed, but diagnostic advances and greater awareness have led to a broader understanding of its epidemiology and clinical spectrum.

**Objective:**

To describe the evolving clinical, laboratory, and histological profile of Brazilian adults diagnosed with CD over the past five decades.

**Methods:**

A retrospective descriptive study was conducted including 181 adults with biopsy-confirmed CD managed at a referral private practice in in Curitiba, Brazil, from 1975 to 2025. Patients were stratified into three diagnostic phases: phase 1 (1975-2000), phase 2 (2001-2010), and phase 3 (2011-2025). Clinical manifestations, nutritional status, comorbidities, histological findings, and laboratory parameters were analyzed comparatively.

**Results:**

The cohort was predominantly female (84.8%), with a median age of 36 years at diagnosis. Marsh III lesions were observed in 98.0% of phase 1 cases, remaining the most frequent finding across phases. Underweight was prevalent in phase 1 (63.2%) but declined significantly in phase 2 (26.4%) and phase 3 (16.5%), reflecting, probably, earlier detection. Diarrhea predominated in phases 1 and 2, whereas constipation became more frequent in phase 3. Extraintestinal manifestations such as anemia, fatigue, and weight loss decreased over time, while reports of comorbid immune-mediated diseases and family history of gluten-related disorders increased.

**Conclusion:**

Over the last 50 years, the clinical presentation of CD in Southern Brazil has shifted from advanced malnourished states with diarrhea to more heterogeneous profiles including constipation and overweight. Despite advances in serology, duodenal biopsy remains the gold standard for diagnosis. These findings underscore the importance of continuous clinical vigilance and reflect a sustained contribution to CD management in Brazil.

## HIGHLIGHTS

• Clinical presentation of celiac disease in Brazil shifted over five decades.

• Duodenal biopsy with Marsh classification remains the diagnostic gold standard.

• Diarrhea declined, while reflux and bloating became more common.

• Serological screening and awareness enabled earlier, less severe diagnoses

## RESUMO

**Contexto:**

Desde meados do século XX, a doença celíaca (DC) deixou de ser considerada predominantemente uma condição pediátrica para ser progressivamente reconhecida em todas as faixas etárias. No Brasil, a DC foi inicialmente subdiagnosticada, mas os avanços diagnósticos e o aumento do conhecimento ampliaram a compreensão de sua epidemiologia e espectro clínico.

**Objetivo:**

Descrever a evolução do perfil clínico, laboratorial e histológico de adultos brasileiros diagnosticados com DC ao longo das últimas cinco décadas.

**Métodos:**

Estudo retrospectivo descritivo incluindo 181 adultos com diagnóstico confirmado por biópsia de DC, acompanhados em clínica privada de referência em Curitiba, Paraná, Brazil, entre 1975 e 2025. Os pacientes foram estratificados em três fases diagnósticas: Fase 1 (1975-2000), Fase 2 (2001-2010) e Fase 3 (2011-2025). Foram analisadas comparativamente manifestações clínicas, estado nutricional, comorbidades, achados histológicos e parâmetros laboratoriais.

**Resultados:**

A coorte foi composta predominantemente por mulheres (84,8%), com idade mediana de 36 anos ao diagnóstico. Lesões Marsh III foram observadas em 98,0% dos casos da Fase 1, permanecendo como o achado mais frequente nas fases subsequentes. O baixo peso foi prevalente na Fase 1 (63,2%), mas diminuiu significativamente na Fase 2 (26,4%) e Fase 3 (16,5%), provavelmente devido ao diagnóstico mais precoce. A diarreia predominou nas Fases 1 e 2, enquanto a constipação tornou-se mais frequente na Fase 3. Manifestações extraintestinais como anemia, fadiga e perda de peso reduziram-se ao longo do tempo, enquanto relatos de doenças autoimunes associadas e histórico familiar de distúrbios relacionados ao glúten aumentaram.

**Conclusão:**

Nos últimos 50 anos, a apresentação clínica da DC no Sul do Brasil evoluiu de quadros avançados com desnutrição e diarreia para perfis mais heterogêneos, incluindo constipação e sobrepeso. Apesar dos avanços sorológicos, a biópsia duodenal permanece o padrão-ouro diagnóstico. Esses achados ressaltam a importância da vigilância clínica contínua e refletem uma contribuição consistente para o manejo da DC no Brasil.

## INTRODUCTION

Since 1950, when a Dutch pediatrician Willem-Karel Dicke demonstrated that many children with celiac disease (CD) could be successfully treated with a diet free of wheat and rye flours, the disorder was primarily regarded as a pediatric condition and was rarely diagnosed in adults^1^. However, advancements in diagnostic methods, along with growing awareness and reports of increasing prevalence, have contributed to the earlier and more frequent detection of CD across all age groups^2^. In Brazil, celiac disease (CD) remained relatively unknown for much of the past seventy years. Initial reports focused on pediatric cases, followed by increasing recognition of the disease in adults^4^ ^,^ ^5^. Currently, CD is recognized as a condition that can affect individuals of all ages in Brazil, including the elderly^6^.

To illustrate trends in the clinical presentation of celiac disease (CD) over time, we describe distinct phases of our experience with adult patients, incorporating personal observations with publications and aligning with the chronological classification of CD onset proposed by Tommasini et al.^7^. During this initial period (phase 1 - 1975-2000), CD diagnosis was based exclusively on clinical features and confirmed by histological examination of small bowel biopsies. Histopathological findings included villous atrophy, epithelial surface changes, mucosal thickening, and glandular hypertrophy, as originally described by Shiner in 1960^8^. Biopsy specimens were obtained using the Crosby-Kugler capsule, a simple but reliable tool for routine small bowel sampling^9^ ^,^ ^3^. From 1972 onward, intraepithelial lymphocyte (IEL) counts were incorporated into our diagnostic routine to help distinguish CD from other enteropathies^10^ ^,^ ^11^. Starting in 1982, upper gastrointestinal endoscopy (UGIE) replaced the capsule method, allowing for more precise and less invasive biopsy collection^12^ ^,^ ^13^. From 1972, histological interpretation was guided by the original Marsh classification^14^, and archived biopsy samples from earlier periods were retrospectively reviewed under these updated criteria.

The availability of serological testing progressively enhanced our diagnostic capacity. Initial detection focused on anti-gliadin antibodies (AGA-IgA and AGA-IgG)^15^ and anti-reticulin antibodies^16^ ^,^ ^17^, after ensuring normal total IgA levels. In 1984, Chorzelski reported anti-endomysium antibodies (AEA)^18^, which we began to test using umbilical cord tissue as a substrate from 1997, in line with the method proposed by Volta et al.^5^ ^,^ ^19^. The subsequent development of ELISA for anti-tissue transglutaminase antibodies (anti-TTG)^20^ provided a more practical and widely used alternative^21^. More recently, tests for deamidated gliadin peptides (DGP), although primarily used in pediatric populations, have been adopted in select cases to monitor adherence to a gluten-free diet (GFD)^22^.

Initial HLA typing in CD identified the presence of HLA-A1 and HLA-B8^23^, and was subsequently confirmed in our cases^24^. Later, the association of CD with HLA-DQ2 and/or HLA-DQ8, as described by Sollid et al. became more widely recognized^25^. However, HLA-DQ2/DQ8 typing was not routinely performed and remained primarily reserved for cases with diagnostic uncertainties or for screening relatives of patients with confirmed CD.

During the past 20 years, many changes occurred in the human social behavior in all the world with reflex on alimentary diet costumes. Some points to call attention are the little spending time on food preparation at home that is essential to healthier dietary habits with consequent outside excessive fast-food and ultra-processed foods consumption that increases obesity^26^ ^,^ ^27^. It is to expect that persons that not know they have CD could manifest symptoms different from the referred by patients in phase 1 of the study (1975-2000). Our experience attested these facts.

In 2019 COVID-19 pandemic occurred with a deep impact on all disorders, including CD. On COVID-19 lockdown, since march 2020, we used telemedicine to orient patients^28^ ^,^ ^29^ and to pass crucial informations^30^ ^-^ ^32^. No new cases were diagnosed in this year. Since 2021 to nowadays new cases were diagnosed using the same protocol to compare the findings of CD at diagnosis with the anterior phases and in accordance to the more recent guidelines^33^ ^,^ ^34^.

## OBJECTIVE

The objective of this study is to present the evolving clinical profile of a large cohort of Brazilian adults diagnosed with CD, through a comparative analysis of clinical, laboratory, and histological findings from 1975 to 2025.

## METHODS

### Design and ethical issues

This was a retrospective descriptive study approved by the local Research Ethics Committee (protocol number 39920920.2.0000.0103). The study adhered to the principles of the Declaration of Helsinki and followed Good Clinical Practice guidelines. Data collection was performed through a comprehensive review of medical records from patients seen between 1975 and May 2025. All patients were managed by the same physician at a referral gastroenterology private practice in Curitiba, Paraná, Brazil.

### Inclusion and exclusion criteria

Eligible participants were those with a clinical diagnosis of CD based on characteristic signs and symptoms, with diagnostic confirmation through duodenal biopsy demonstrating histopathological changes consistent with the original Marsh classification^14^. Patients with incomplete or insufficient medical records, patients in a gluten-free diet (GFD) or with diagnosis of other gastrointestinal disorders were excluded.

### Data collection

Participants were stratified into three diagnostic phases: phase 1 (1975-2000), phase 2 (2001-2010) and phase 3 (2011-2025).

Based on our observed changes in clinical presentation at diagnosis, after the phase 1 we divided the subsequent experience in phase 2 (2001 to 2010) and phase 3 (2011 to 2025). During both phases, the determination of antibodies remained^5^ and UGIE, with histopathological analysis of biopsy samples using the Marsh classification, was consistently performed throughout^14^ ^,^ ^33^ ^,^ ^34^. HLA DQ2/DQ8 testing has been reserved for cases with discrepancies in diagnosis, in seronegative cases^35^ and for diagnosing CD in relatives of patients^23^.

Patients across the three phases were compared regarding trends in age at diagnosis, frequency of general and gastrointestinal complaints, presence of extraintestinal manifestations and CD related complications. Nutritional status was assessed through weight and height measurements. Reported comorbidities and family history of gluten-related disorders were also analyzed. Histopathological findings from duodenal biopsies were compared according to the original Marsh classification^14^.

### Data analysis

Data was collected in frequency tables. Nominal data were expressed in percentages. The central tendency of numerical data was expressed as means and standard deviation (SD) if the distribution was normal and as median and interquartile range (IQR) if the distribution was nonparametric. Comparisons of BMI, age, and diagnostic delay between phases were performed using the unpaired t-test and Mann Whitney test and the Marsh classification by using the chi-squared test. The adopted significance was 5%.

## RESULTS

**FIGURE 1:**
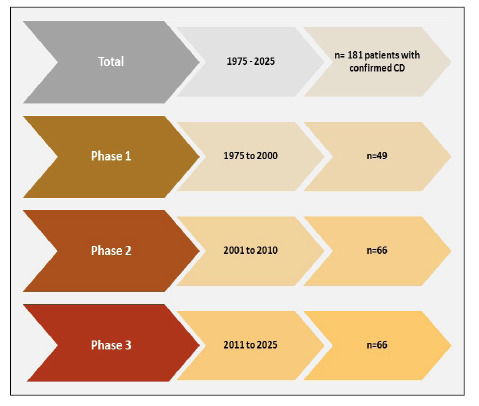
Phases of study and number of patients with CD studied.

A total of 181 adults consuming gluten and diagnosed with biopsy-confirmed celiac disease (CD) were evaluated at a private clinic in Curitiba, Paraná, Brazil, between 1975 and 2025. The participants stratified in the three diagnostic phases were illustrated in Figure 1 **,** as phase 1 (1975-2000, n=49), phase 2 (2001-2010, n=66) and phase 3 (2011-2025, n=66).

The cohort was predominantly composed of females (84.8%) and the median age at diagnosis was 36.0 years (interquartile range: 26-46), ranging from 18 to 69 years, with no significant differences between sexes.

Table 1 presents the demographic characteristics of the studied patients and the histopathological findings from duodenal biopsies. The age of symptom onset showed no significant differences throughout the study period. Duodenal biopsies classified as Marsh III lesions, indicating more severe mucosal damage, were observed in 98.0% of patients in phase 1 and remained the most frequent finding in the subsequent phases. Moreover, the number of patients with underweight (BMI) was observed in 63.2% of cases in phase 1 and decreased significantly in phase 2 (26.4%) and phase 3 (16.5%).

**TABLE 1:** Clinical data from studied patients.

	Total		1975-2000		2001-2010		2011-2025		P*	P**
	(n=181)		(n=49)		(n=66)		(n=66)			
	N	%	N	%	N	%	N	%		
**Sex**									0.78	0.90
Female	153	(84.8)	40	(81.6)	57	(86.4)	56	(84.8)		
Male	28	(15.2)	9	(18.4)	9	(13.6)	10	(15.2)		
**Age at diagnosis**									0.12	0.56
Median (IQR)	36.0	(26-46)	33.5	(27-47)	33.0	(22-45)	39.0	(26-47)		
**Body mass index (BMI) Kg/m** ^2^									0.001	0.58
Underweight	48	(16.5)	24	(63.2)	14	(26.4)	10	(15.1)		
Normal	88	(48.6)	12	(31.6)	31	(58.5)	45	(68.2)		
Overweight	16	(8.9)	2	(5.3)	7	(13.2)	7	(10.6)		
Obesity	5	(2.8)	0	(0.0)	1	(1.9)	4	(6.1)		
**Duodenal Byopsy Classification - Marsh**									0.10	0.88
I	12	(7.0)	0	(.0)	7	(10.6)	5	(7.6)		
II	14	(7.4)	1	(2.0)	7	(10.6)	6	(9.1)		
III	155	(85.6)	48	(98.0)	52	(78.8)	55	(83.3)		

* Comparison between the three phases. **comparing 2001-2010 vs 2011-2025. IQR=interquartile range.

Table 2 presents the gastrointestinal symptoms reported by the study participants. Across all phases, the most frequently observed symptoms were flatulence, abdominal distension, and borborygmi. Diarrhea, traditionally considered a hallmark manifestation of CD, was more frequent in phase 1 and phase 2, whereas constipation emerged as an increasingly frequent complaint in phase 3.

**TABLE 2:** Gastrointestinal symptoms observed in patients with celiac disease during the study periods.

	Total		1975-2000		2001-2010		2011-2025		P*	P**
	(n=181)		(n=49)		(n=66)		(n=66)			
	N	%	N	%	N	%	N	%		
Flatulence	139	(76.8)	44	(95.7)	46	(71.9)	49	(74.2)	0.002	1.0
Abdominal distension	131	(72.4)	42	(93.3)	45	(69.2)	44	(66.6)	0.001	0.56
Borborigmy	119	(65.7)	39	(90.7)	32	(50.8)	48	(72.7)	<0.0001	0.023
Dhyarreia	112	(61.9)	42	(87.5)	46	(70.8)	24	(36.3)	<0.0001	<0.0001
Abdominal pain	98	(54.1)	32	(71.1)	34	(54.0)	32	(48.5)	0.11	0.85
Gastroesofaghic reflux	82	(45.3)	15	(46.9)	26	(40.6)	41	(62.1)	0.10	0.044
Apthas	52	(34.4)	8	(25.0)	27	(42.2)	17	(25.7)	0.21	0.25
Epigastric pain	46	(25.4)	8	(26.7)	17	(26.6)	21	(31.2)	0.73	0.54
Dyspepsia	44	(24.3)	13	(40.6)	15	(23.4)	16	(24.2)	0.11	0.82
Nausea	39	(21.5)	16	(40.0)	12	(18.8)	11	(16.6)	0.012	0.62
Vomit	32	(17.6)	12	(28.6)	8	(12.5)	12	(18.2)	0.11	0.32
Constipation	26	(14.4)	5	(10.4)	4	(6.3)	17	(25.7)	0.035	0.012
Dysgeusia	8	(4.9)	0	(.0)	4	(6.6)	4	(7.8)	1.0	1.0

*Comparison between the three periods.**comparing 2001-2010 vs 2011-2025.

Table 3 presents the extra-digestive complaints and complications associated with CD. In phase 1, significantly higher frequencies of weight loss (82.9%), anemia (80.0%), and fatigue (75.0%) were observed. The prevalence of other immune-mediated disorders and the number of relatives with gluten-related conditions reported by patients increased significantly in phase 2 and phase 3.

**TABLE 3:** Extra digestive complaints and complications found in patients with celiac disease during the study periods.

	Total		1975-2000		2001-2010		2011-2025		P*	P**
	(n=181)		(n=49)		(n=66)		(n=66)			
	N	%	N	%	N	%	N	%		
Anemia	76	(41.1)	32	(80.0)	25	(41.0)	19	(28.8)	<0.0001	0.16
Under height	4	(2.2)	2	(6.5)	2	(3.3)	0	(.0)	0.20	0.50
Weight gain	26	(14.4)	4	(9.5)	9	(15.0)	8	(12.1)	0.43	0.58
Weight loss	79	(43.6)	34	(82.9)	24	(40.0)	21	(31.8)	<0.0001	0.84
Osteopenia ^+^	-		-		18	(32.1)	19	(36.4)	0.28	0.58
Osteoporosis ^+^	-		-		18	(32.1)	5	(7.5)	0.017	0.005
Anxiety	71	(42.0)	24	(53.3)	15	(23.4)	32	(48.5)	<0.0001	0.002
Depression	62	(39.5)	16	(34.8)	25	(47.2)	21	(31.8)	0.39	0.31
Astenia	53	(39.1)	30	(75.0)	6	(11.1)	17	(25.8)	<0.0001	0.001
Other autoimmune disease	40	(21.2)	2	(4.1)	18	(27.7)	20	(30.1)	0.001	0.90
Relative with celiac disease	42	(26.8)	6	(13.0)	13	(25.0)	23	(34.8)	0.007	0.002

*Comparison between the three periods. **comparing 2001-2010 vs 2011-2025. ^+^laboratory methods for the assessment of bone alterations available only after the year 2000.

## DISCUSSION

In this retrospective study, trends in the clinical presentation of CD over the past 50 years were analyzed in a large cohort of biopsy-proven adult patients followed in a Brazilian private practice. It is important to emphasize that our clinical experience and resulting publications have aligned with advances in diagnostic tools for CD, including shifts in the age at diagnosis, as previously described by Tommasini et al.^7^.

In phase 1 (1975 to 2000), only 49 adult cases of CD were diagnosed over a 25-year period, highlighting the lack of clinical suspicion for the condition at that time. In contrast, 122 patients were diagnosed in the subsequent 25 years, reflecting increased awareness among physicians-including non-gastroenterology specialists-as well as the broader availability of serologic testing and the more widespread use of upper gastrointestinal endoscopy (UGIE) with duodenal biopsies^33^ ^,^ ^34^.

Regarding gender distribution, all phases showed a predominance of females over males. A similar pattern was observed for age at diagnosis, which remained consistent across all cases, with a median age of 35.5 years, equally distributed between women and men, as reported in several publications^33^ ^,^ ^34^. As this study was conducted in Southern Brazil, a region with strong European ancestry, the findings are consistent with those reported in similar populations^5^ ^,^ ^33^ ^,^ ^34^.

There were no differences in patient height across the study phases, even though individuals in phase 1 were more frequently undernourished. However, underweight status was notably prevalent in the first period, indicating diagnosis at more advanced stages of the disease, as confirmed by Marsh III lesions in 98.0% of cases. This may be attributed to longer diagnostic delays^33^ ^,^ ^34^. Over time, the detection of overweight and obesity became more common, reflecting changes in dietary habits^26^ ^,^ ^27^. When comparing phase 2 and phase 3, the prevalence of overweight remained stable, but obesity rates increased nearly fourfold, similar to findings reported by Maleki et al., likely due to greater consumption of fatty foods, sugars, proteins, and high-calorie beverages^36^.

Regarding the histology, in accordance with Singh et al.^37^, it is probable that CD can cause atrophy of the intestinal mucosa independently of body weight and that overweight is not a factor that prevents villous atrophy. However, it is possible that individuals with overweight or obesity present with milder or less evident symptoms, which may hinder diagnosis. The same authors also reported no difference in the severity of villous atrophy among patients who were underweight, of normal weight, or overweight^37^. Additionally, in relation to the finding of mild enteropathy (Marsh I or II), present in phase 2 and phase 3, it is important to highlight that patients with mild enteropathy experienced the same clinical manifestations as those with Marsh III. These findings were similar to those reported by Zanini et al. since 2013^38^. The authors suggest that, despite changes in the mode of presentation and the availability of new diagnostic tools, small bowel mucosal biopsy has remained the gold standard for CD diagnosis to this day^33^ ^-^ ^34^.

The more reported GI symptoms were flatulence, abdominal distention and borborygmus in all the phases, as pointed out by several studies by different authors of distinct countries^33^ ^,^ ^34^. Diarrhea reported as the meanly complaint of patients with CD was referred more in phase 1 and phase 2*,* as constipation called attention in phase 3, probably consequence of changes in the diet. The comparison of cases with and without diarrhea showed no difference in age or gender. Nausea was more reported in phase 1. Gastroesophagic reflux appeared in 2/3 of the cases in phase 3 in accordance with reports in gastroenterological clinics^39^. All the others complaints did not present statistical differences.

Findings from phase 1 revealed significantly higher rates of weight loss, anemia, and fatigue, indicating that patients were diagnosed at more advanced stages of CD. In subsequent years, particularly in phase 3, there was a significant decline in the frequence of weight loss and fatigue, aligning with the trends reported by Maleki et al.^36^. Anxiety and depression remained relatively constant across all phases, even in pandemic phase, confirming the observations of Bascuñán et al. in Chile^40^ and Falcomer et al in Brazil^41^. Bone disease began to be systematically evaluated in our patients only after 2001, with the implementation of DEXA scanning. While the prevalence of osteopenia remained relatively stable in recent years, osteoporosis was more frequent in phase 2.

In our protocol, questions regarding immune-mediated diseases (IMDs) in patients were incorporated after 2001 and showed similar prevalence across the subsequent phases. Reports of IMDs and gluten-related disorders among relatives became more frequent in the later phases, likely due to increased diagnostic efforts by family healthcare providers and greater public awareness driven by media coverage.

This study has some limitations inherent to its retrospective design. Although all patients were evaluated using a standardized clinical, laboratory, and histological protocol, a primary source of bias stems from the fact that all data were derived from a single referral private practice. A major strength of this study lies in the fact that all patients were evaluated by the same physician, ensuring consistency in clinical assessment and drawing upon five decades of continuous expertise in CD through clinical practice and research. Over this extensive period, our group conducted numerous investigations, culminating in presentations at national and international conferences, as well as participation in lectures and specialty meetings. Several of these studies focused on populations at increased risk: relatives of patients^5^, individuals with Down syndrome^42^ and patients with immune related disorders (as type 1 diabetes^43^, thyroid diseases^44^). In addition, particular attention was given to comorbidities commonly associated with CD, such dermatitis herpetiformis^45^, metabolic bone disease^46^ and reproductive aspects^47^. Recently, particular emphasis has been placed on reporting our group’s experience in managing male patients with CD, a topic that remains underrepresented in the literature^48^. Collectively, these studies have made a significant contribution to advancing both the diagnosis and clinical management of CD in our region.

## CONCLUSION

Our data illustrate changes in the clinical presentation of CD over the studied period. Flatulence, abdominal distension, and borborygmi remain the predominant symptoms, whereas the frequency of diarrhea has significantly declined in recent years. The implementation of serological screening with disease-specific biomarkers has facilitated earlier diagnosis. Upper gastrointestinal endoscopy with duodenal biopsies remains the gold standard for diagnostic confirmation, with Marsh III histological findings being the most frequently observed. Nevertheless, milder forms of enteropathy (Marsh I and Marsh II) are increasingly identified in early-stage diagnoses. The findings presented herein, derived from five decades of clinical and research activity in Southern Brazil, provide a real-world perspective from a reference gastroenterology clinic. This body of work reflects not only sustained dedication to patient care and clinical practice but also long-standing scientific contributions that are in line with the most relevant international literature on CD.

## Data Availability

Data-available-upon-request
